# P-1683. Evaluation of Outpatient Clindamycin Prescriptions Across a Large Military Healthcare Network

**DOI:** 10.1093/ofid/ofae631.1849

**Published:** 2025-01-29

**Authors:** Mark Derasmo, Kayla R Scheps, Joseph Marcus

**Affiliations:** Brooke Army Medical Center, JBSA Ft Sam Houston, Texas; Brooke Army Medical Center, JBSA Ft Sam Houston, Texas; Brooke Army Medical Center, JBSA Ft Sam Houston, Texas

## Abstract

**Background:**

Introduction: While clindamycin has broad Gram-positive and anaerobic activity, its use has been limited by toxicity and increasing resistance. Multiple clinical practice guidelines (CPGs) at Joint Base San Antonio recommend against clindamycin usage due to poor coverage of Gram-positive bacteria in local antibiograms. Despite this recommendation, a significant number of clindamycin outpatient prescriptions are ordered each year. The driver of these prescriptions is unknown. This study describes prescriber, diagnostic, and patient factors contributing to outpatient clindamycin usage at a large military medical system.

**Table 1:**
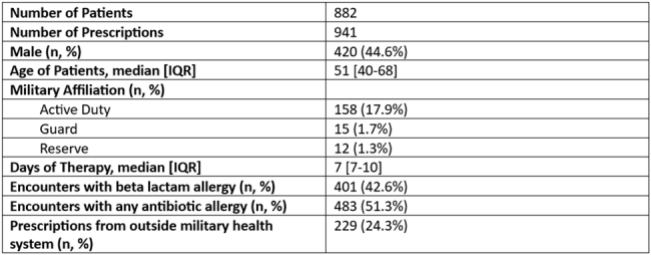
Characteristics of 882 Patients Who Received an Outpatient Clindamycin Prescription at Joint Base San Antonio, 2023

**Methods:**

Outpatient clindamycin prescriptions from January-December 2023 filled at pharmacies on Joint Base San Antonio were evaluated to determine prescription duration, diagnostic indication, age, antibiotic allergies, prescriber, and prescribing clinic. Antibiotic indication was compared between those with beta-lactam allergies to those without beta-lactam allergies by Chi-squared.

**Table 2:**
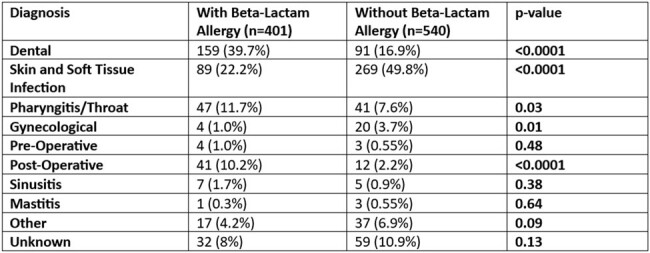
Prescriptions by Diagnostic Group and Beta-Lactam Allergy Status of Patient

**Results:**

During the study period there were 941 prescriptions in 882 patients. The cohort was predominately female (52%) with a median age of 51 [IQR 40-68] (**Table 1)**. Of these scripts, 229 (24%) were written by clinicians outside the military facility. The clinics with the most prescriptions were the Emergency Department (33%), Dental clinics (21%), Primary Care (16%), and Surgical clinics (14%). Patients with beta-lactam allergies were significantly more likely to have a dental indication (39.7% vs. 16.9%, p=< 0.0001) and those without beta-lactam allergies were more likely to have a skin and soft tissue infection (49.8% vs. 22.2%, p=< 0.0001) (**Table 2).** While there were 491 prescribers in this study, ten (2%) clinicians (six from the Emergency Department) prescribed 18% of all clindamycin prescriptions, 67% of which were in the absence of a beta-lactam allergy.

**Conclusion:**

Patterns of outpatient clindamycin use across large health systems have not been well described. This study identifies potential areas for local stewardship interventions that have not previously been considered, including focused CPGs for emergency room clinicians as well as penicillin allergy de-labeling in outpatient dental clinics.

**Disclosures:**

**All Authors**: No reported disclosures

